# Adjuvant concurrent chemoradiation using intensity-modulated radiotherapy and simultaneous integrated boost for resected high-risk adenocarcinoma of the distal esophagus and gastro-esophageal junction

**DOI:** 10.1186/1748-717X-8-33

**Published:** 2013-02-11

**Authors:** Brian P Yaremko, David A Palma, Abigail L Erickson, Gregory Pierce, Richard A Malthaner, Richard I Inculet, A Rashid Dar, George B Rodrigues, Edward Yu

**Affiliations:** 1 Department of Radiation Oncology, Western University, London Regional Cancer Program Room A3-810 Ed and Irene Fregin Building 790 Commissioners Road E, N6A-4L6, London, ON, Canada; 2 Department of Radiation Oncology, Western University, London Regional Cancer Centre, 790 Commissioners Road East, N6A 4L6, London, ON, Canada; 3 Department of Surgical Oncology, Western University, London Regional Cancer Centre, 790 Commissioners Road East, N6A 4L6, London, ON, Canada; 4 Department of Radiation Oncology and Department of Clinical Epidemiology and Biostatistics, Western University, London Regional Cancer Centre, 790 Commissioners Road East, N6A 4L6, London, ON, Canada

**Keywords:** Gastro-esophageal junction, Gastro-oesophageal junction, GEJ, IMRT, Adjuvant, Simultaneous integrated boost, SIB

## Abstract

**Purpose:**

Multimodality therapy leads to improved outcomes for adenocarcinoma of the distal esophagus and gastroesophageal junction (GEJ) over surgery alone. At our institution, adjuvant chemoradiation (chemoRT) using IMRT and SIB is standard of care for resected high-risk disease. In this study, we review our experience with a recent cohort of patients treated in this manner.

**Methods and materials:**

We identified 18 patients with resected T3 and/or N1 adenocarcinoma of the distal esophagus and GEJ who received adjuvant chemoRT. A large elective volume (PTV1) and a smaller high-risk volume (PTV2) were irradiated simultaneously using IMRT and an SIB technique. All patients received concurrent chemotherapy. Relevant clinical outcomes are reported.

**Results:**

The median dose to 95% of PTV1 was 3747cGy and to 95% of PTV2 was 4876cGy. All RT was given in a median of 28 daily fractions. Four patients did not complete chemotherapy. At a median follow up of 952 days from the start of RT, 7 of 18 patients were dead; of these, 3 had developed local recurrence only; 3 had developed both local and distant recurrence; 1 died of a late toxicity, without recurrence. OS was 88% at 1year, 76% at 2 years and 58% at 3 years. Freedom from local recurrence was 88% at 1 year, 82% at 2 years and 82% at 3 years. Freedom from distant recurrence was 72% at 1 year, 67% at 2 years and 56% at 3 years. Toxicity was acceptable.

**Conclusions:**

Adjuvant concurrent chemoRT with IMRT and SIB is feasible for resected high-risk adenocarcinoma of the distal esophagus and GEJ. Our results describe how modern treatment techniques can be employed as part of a treatment paradigm that is neither commonly used nor commonly described in the literature.

## Introduction

The incidence of adenocarcinoma of the distal esophagus, gastro-esophageal junction (GEJ) and proximal stomach is increasing [1]. These cancers can grow to an advanced size before becoming apparent, and they can access an extensive lymphatic network [2], increasing the risk of metastasis and compromising outcome. For such reasons, the prognosis for this disease remains dismal. The mainstay of curative-intent treatment is surgery, where outcome correlates with the completeness of surgical resection [3]. Unfortunately, surgery alone is rarely curative if there is nodal involvement [4], and thus there is a clear need to establish improved mechanisms for delivering adjuvant therapy.

Individually, the benefits of radiotherapy (RT) and chemotherapy beyond surgery are modest [5]. The best outcomes have been achieved with trimodality therapy, where meta-analysis has identified significantly improved overall survival (OS) over surgery alone with the use of induction chemoRT [6-8]. Based upon such results, neoadjuvant chemoRT has become the standard of care for resectable carcinoma of the esophagus and GEJ.

Despite this, adjuvant concurrent chemoRT remains our institutional standard of care for patients with respectable carcinoma of the distal esophagus and/or GEJ. Such patients are taken immediately to surgery, after which those with pathologically proven T3 or N1 disease are offered adjuvant chemoRT. Historically, we delivered such treatment using multiphase 3-dimensional conformal radiotherapy (3DCRT) techniques [9,10]. We have now established intensity-modulated radiotherapy (IMRT) with simultaneous integrated boost (SIB) as our new standard. We review herein our recent clinical experience with this novel technique in a cohort of patients.

## Methods

### Ethics approval

This retrospective review was performed with the approval of our Institutional Health Sciences Research Ethics board.

### Study population

Between 2007 and 2010, a cohort of 18 patients with resected adenocarcinoma of the distal esophagus and GEJ received adjuvant chemoRT. Every patient had pathologic confirmation of T3 and/or N1 disease. Every patient was fit enough to tolerate adjuvant aggressive chemoRT. After treatment, every patient was seen every three months for the first year, and at least every 6 months thereafter. The clinical characteristics of our study cohort are presented in Table 1.

**Table 1:** Patient demographics

**Demographic**	**Subset**	**Value**
general	total number	18
	male	15
	female	3
age (years)	mean	65
	median	63
histology	adenocarcinoma	18
	other	18
stage	T3N0	1
	T3N1	14
	T3N2	2
	other	0

### Surgery

Fifteen patients had video-assisted transhiatal esophagectomy with cervical anastomosis. One patient had transhiatal esophagectomy with right video-assisted mobilization of the esophagus. One patient required trans-thoracic esophagectomy with thoracotomy (three-hole esophagectomy). One patient required open transhiatal esophagectomy with laparotomy. All technically accessible lymph nodes were removed from the lower thoracic and upper abdominal nodal basins. Marking clips were placed to guide adjuvant radiotherapy treatment planning.

### Chemotherapy

Prior to the start of RT, patients received two cycles of epirubicin-cisplatin-5-fluorouracil (ECF) alone (epirubicin: 50 mg/m2; cisplatin: 60 mg/m2; 5FU infusion: 200 mg/m2•d). This was followed by a further two cycles of CF given concurrently with RT (cisplatin: 60 mg/m2; 5FU infusion: 200 mg/m2•d). Each cycle was 3 weeks in duration, with the understanding that any cycle(s) could be given at reduced dose (or withheld altogether) at the discretion of the treating Medical Oncologist.

### Radiotherapy treatment planning

#### General

All patients went for CT simulation during cycle 2 of chemotherapy. Prior to 2008, free-breathing CT simulation scans were standard. From 2008 onward, we used 4-dimensional CT (4DCT) to incorporate respiratory motion. Motion management was established using the Real-Time Position Management™ (RPM™) system (Varian Incorporated, Palo Alto, CA). All patients were immobilized with arms up, in a vacuum fixation device. Intravenous and oral contrast agents were not used.

#### Delineation of ICRU target volumes

We defined two clinical target volumes (CTV). Lower-dose volume CTV1 covered nodal basins at risk of harboring metastatic disease: the celiac axis, porta hepatis and splenic hilar nodes in the abdomen, and levels 1 through 8 in the mediastinum. In all cases, the superior border of CTV1 was set 3 cm superior to the anastomosis. Boost volume CTV2 covered the pre-surgical location of the primary disease, any bulky adenopathy and sites of close or positive margins. In general, CTV2 included the lower mediastinum and upper abdomen. For a single patient, CTV2 also included the anastomosis because of a close longitudinal surgical margin.

Prior to 2008, we applied margins of 1 cm superiorly-inferiorly and 5 mm radially to CTV 1 and CTV2 to account for tumor motion. From 2008 onwards, once 4DCT became standard, we delineated CTV1 and CTV2 on both the full-expiration and full-inspiration images to account for respiratory motion. We then added an isotropic margin of 3 mm to account for residual motion, defining internal target volumes (ITV) ITV1 and ITV2. Finally, we added a 5 mm isotropic margin around ITV1 and ITV2 for setup, defining planning target volumes (PTV) PTV1 and PTV2.

**Figure 1:**
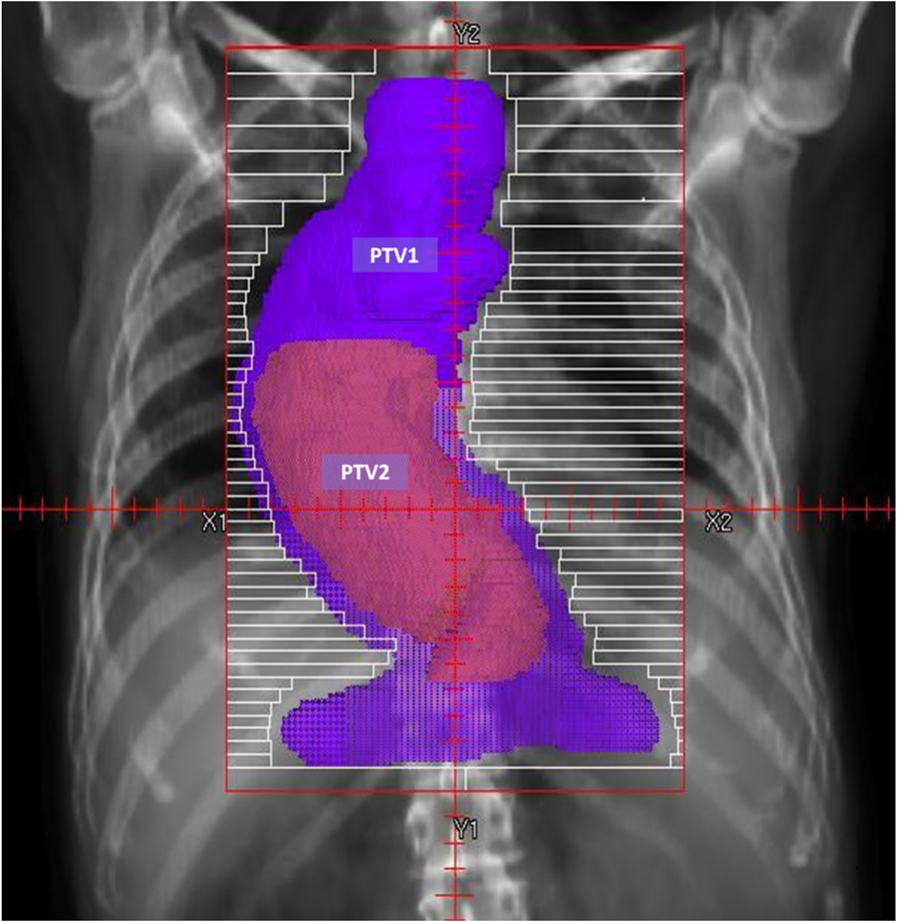
**Beam’s**-**eye View: Target Volumes for IMRT in a 65 year-old male with resected T3N1 adenocarcinoma of the distal esophagus and GEJ.** PTV1, encompasses abdominal nodes, mediastinal nodes and anastomosis. PTV2, covers the pre-operative site of disease and any close or positive surgical margins.

Figure 1 depicts a typical example of PTV1 and PTV2.

#### Prescription dose

Historically, we treated these patients in two phases using 3DCRT: in phase 1, a large elective volume, including the anastomosis, was treated to 3600 cGy in 20 daily fractions. Our decision to include the anastomosis was rooted in RTOG 8501, where essentially the entire mediastinum was treated to 3600 cGy in 20 fractions [11]. In phase 2, the high-risk boost volume was treated to 1440 cGy in 8 daily fractions (5040 cGy in 28 fractions total). Radiation portals for the phase 2 boost were defined according to pre-operative imaging and pathology [9].

PTV1 and PTV2 represent the modern cognates of our previous technique [10]. PTV1 and PTV2 were treated using a simultaneous integrated boost (SIB) technique. All treatment was delivered using IMRT. PTV1 was prescribed a nominal dose of 3900 cGy in 28 daily fractions, representing a biologically equivalent dose to our historical standard of 3600 cGy in 20 fractions. PTV2 was, for most patients, prescribed a nominal dose of 5040 cGy in 28 daily fractions; two patients were prescribed 5400 cGy in 30 daily fractions, with the higher dose selected because of close surgical margins at the original tumor location.

#### Organs at risk

Dose to normal tissues was limited according to published standards [12,13]. For the lung, we defined an evaluable lung volume as the total lung less any overlap with CTV1 (TL-CTV1). This volume was typically limited to 20 Gy to ≤30%, and a mean dose of 18 Gy or less.

### Dosimetric comparison

Because our new technique represents a revision of our previous technique, a dosimetric comparison of our historical cohort and our current cohort would be instructive. Unfortunately, our historical cohort was planned using either an outdated treatment planning system (now decommissioned), or using plain-film radiography. Either way, a detailed dosimetric analysis of our original cohort is no longer possible. However, it is possible to re-plan our modern cohort using our previous technique [9]. We performed this comparison for all 18 cases. The 3DCRT plan was prescribed to 3600 cGy in 20 fractions to PTV1, followed by a sequential boost of 1440 cGy in 8 fractions to PTV2; the IMRT-SIB plan was planned to 3900 cGy to PTV1 and 5040 cGy to PTV2, given concurrently in 28 fractions via an SIB technique. For the purpose of this comparison, the prescription isodose for the 3DCRT plan was adjusted until the dose covering 95% of PTV2 (the D95) was the same as that for the IMRT-SIB plan. Corresponding dosimetric data were generated, and a statistical comparison was performed via a 2-sided, paired t-test.

## Results

### Treatment delivered

RT was initiated at a median of 120 days after surgery. The majority of patients (14 of 18) completed all prescribed RT. For 3 of the remaining 4 patients, 21, 24 and, 26 of the intended 28 fractions were delivered; for the fourth patient, 29 of the intended 30 fractions were delivered. The median delivered number of fractions was 28. Both PTV1 and PTV2 were adequately covered. As-treated dosimetric results are presented in Table 2.

**Table 2:** Resultant dosimetry of clinically treated cohort

**Metric**	**Mean**	**Stdev**	**Median**
intended fractions	28	0.6	28
delivered fractions	27	2.0	28
volume PTV1 (cm3)	1806	506.8	1663
volume PTV2 (cm3)	306	221.8	281
PTV2 / PTV1	0.17	0.10	0.14
PTV1: mean dose (cGy)	4167	310.7	4207
PTV1: D95^*^ (cGy)	3604	320.8	3747
PTV2: mean dose (cGy)	4954	364.1	5074
PTV2: D95^*^ (cGy)	4780	332.6	4876

^*^D95: dose delivered to ≥ 95% of reference volume PTV1 or PTV2.

### Survival

**Figure 2:**
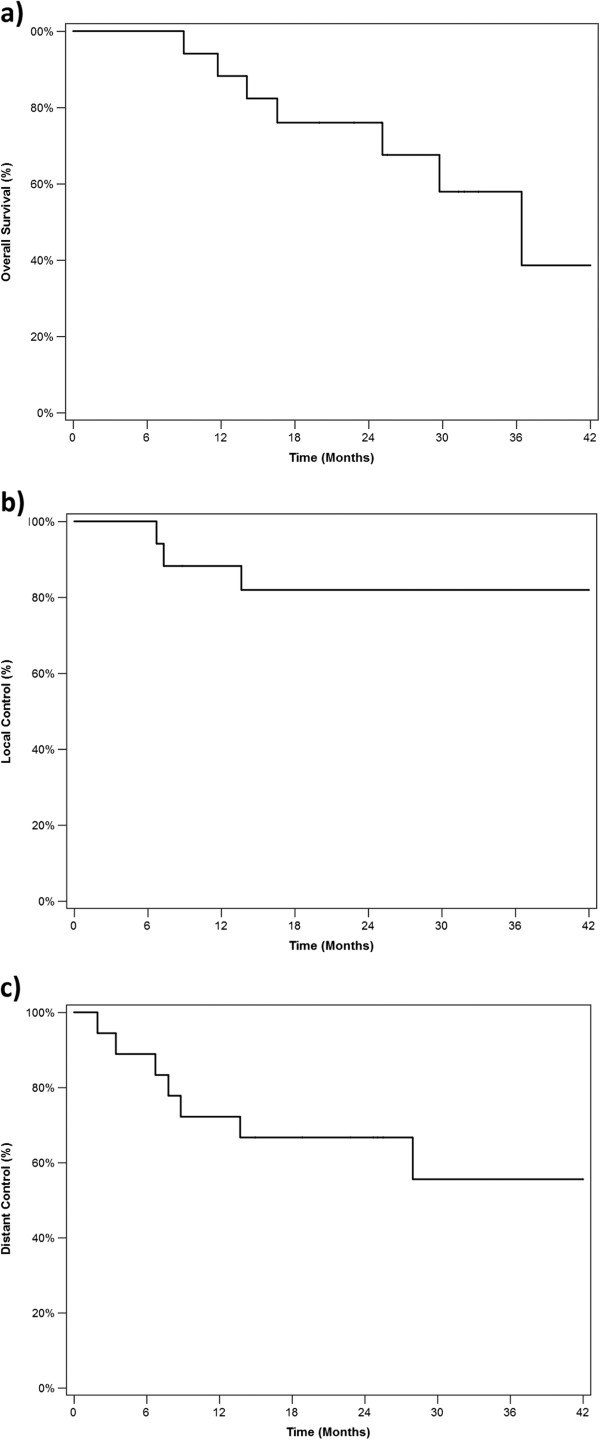
**Kaplan**-**Meier survival curves for (a) overall survival; (b) local recurrence; and (c) distant recurrence (full dataset, n = 18).** Analysis was performed at a median follow up of 952 days after start of RT (or 1065 days from the date of surgery). Overall survival was 88% at 1 year, 76% at 2 years and 58% at 3 years. Freedom from local recurrence was 88% at 1 year, 82% at 2 years and 82% at 3 years. Freedom from distant recurrence was 72% at 1 year, 67% at 2 years and 56% at 3 years.

Analysis was performed at a median follow up of 952 days from the start of RT (or 1065 days from the date of surgery). Recurrences were defined as the earliest instance of: radiographic evidence; biopsy evidence; or abnormality on direct clinical examination. Kaplan-Meier survival curves are presented in Figure 2. Estimated overall survival (OS) was 88% at 1 year, 76% at 2 years and 58% at 3 years. Median OS was 3.0 years. Freedom from local recurrence (LR) was 88% at 1 year, 82% at 2 years and 82% at 3 years. Freedom from distant recurrence (DR) was slightly worse, 72% at 1 year, 67% at 2 years and 56% at 3 years. Median LR-free survival and DR-free survival were not reached. At the time of analysis, 7 of 18 patients were dead: 3 deaths occurred in patients who experienced both LR and DR; 3 deaths were in patients who experienced only DR; a single patient died of a late toxicity (discussed below) without experiencing either LR or DR. Three of 7 deaths were in patients who had not completed the prescribed course of chemoRT. Every patient who experienced LR also experienced DR.

### Toxicity

#### General

Toxicity was graded retrospectively using the Common Toxicity Criteria for Adverse Events (CTCAE) version 4.03 [14].

#### Acute toxicity

For the purposes of this review, “acute” was defined as ≤ 180 days from the start of RT.

Nine of 18 patients experienced acute esophageal toxicity. There were five cases of acute esophagitis (grade 1: n = 4; grade 2: n = 1), occurring at a median of 26 days after starting RT. There were 4 cases of acute stricture requiring dilatation (grade 3), occurring at a median of 37 days after starting RT. For 3 of these, dilatation occurred 36, 37 and 41 days after starting RT; for the fourth, dilatation occurred 86 days *prior to* the start of RT (or 121 days after surgery). One of these patients subsequently developed acute tracheo-esophageal fistula (TEF) 54 days after starting RT, requiring early discontinuation of RT (26 of 28 fractions completed) and urgent surgical repair. The affected patient was free of recurrence at the time of this analysis (238 days after start of RT).

Six of 18 patients experienced acute pulmonary toxicity. There were 2 cases of acute aspiration pneumonitis, occurring 36 days (grade 2) and 97 days (grade 1) after the start of RT (or 167 and 218 days after surgery). Four patients experienced acute grade 1 non-aspiration pneumonitis, without evidence of aspiration on imaging, occurring at a median of 35 days after starting RT.

There were 3 cases of acute grade 1 nausea, 2 cases of acute grade 2 nausea, and 2 cases of acute grade 3 nausea, occurring at a median of 0 days prior to the start of RT. In other words, 4 patients already had established nausea prior to initiation of RT.

Fourteen of 18 patients completed all 4 cycles of chemotherapy. Of the 4 patients who did not, 3 completed 3 cycles, while 1 patient (age 86) completed 1 only cycle before stopping voluntarily (this same patient also voluntarily discontinued RT after only 21 of 28 prescribed fractions). Thirteen of 18 patients experienced acute neutropenia, occurring at a median of 20 days *prior to* the start of RT (grade 1: n = 2; grade 2: n = 7; grade 3: n = 3; grade 4: n = 1). All 18 patients experienced acute lymphopenia (grade 1: n = 0; grade 2: n = 1; grade 3: n = 6; grade 4: n = 11), occurring at a median of 32 days *prior to* the start of RT. Fifteen of 18 patients experienced acute thrombocytopenia (grade 1: n = 8; grade 2: n = 6; grade 3: n = 1; grade 4: n = 0), occurring at a median of 13 days after the start of RT. In total, 14 patients required either a 1-week delay in at least 1 chemotherapy cycle or a dose-reduction of at least 1 chemotherapy agent.

#### Late toxicity

Three patients experienced clinically significant late toxicity. One patient developed grade 5 (fatal) aspiration pneumonitis 357 days after the start of RT. This occurred several months after the patient developed both LR and widespread DR. The patient was debilitated and frail and died only 1 day after aspirating. A second patient experienced esophageal stricture 528 days after starting RT. This patient did not experience acute stricture and responded very well to dilatation. At the most recent follow up assessment, the patient was alive and free of recurrence (966 days after the start of RT). A third patient experienced TEF and grade 5 aspiration pneumonitis 761 days after the start of RT. This patient required multiple dilatations, before, during and after chemoRT and eventually required stricturoplasty, 18 months after the start of RT. Approximately 7 months after stricturoplasty, this patient experienced an acute breakdown of both the transposed esophagus and trachea, leading directly to aspiration and death. At the time of death, the patient‘s disease was under control, with no clinical evidence of LR or DR.

We identified 11 cases of late lymphopenia, with mildly decreased lymphocyte count persisting at a median of 319 days after start of RT. For 3 of these patients, lymphocyte counts eventually returned to normal levels. For the remaining 8 patients, lymphocyte counts remained persistently yet mildly decreased on all subsequent follow-up. We did not identify any late neutropenia or late thrombocytopenia. We did not identify any late nausea.

### Dosimetric comparison

The results of our dosimetric comparison of IMRT-SIB and 3DCRT for the full cohort are presented in Table 3. For normal lung (i.e. total lung less CTV1), the use of IMRT-SIB enabled a significant reduction in V20 and MLD; however IMRT-SIB was associated with a significant increase in V10 compared to 3DCRT. IMRT-SIB also enabled significantly reduced dose to the liver, heart, and cord, along with a non-significant reduction in dose to the kidneys.

**Table 3:** **Dosimetric comparison within the same cohort**: **IMRT**-**SIB technique versus classic 3DCRT technique**

**Metric**	**IMRT**-**SIB**	**3DCRT**	**p (2-sided t-test)**
V20 (TL-CTV1) (−)	0.30	0.40	< 0.005
V10 (TL-CTV1) (−)	0.68	0.54	< 0.005
mean dose (TL-CTV1) (cGy)	1616.9	1760.4	0.070
liver mean dose (cGy)	1748.3	1919.2	0.10
heart mean dose (cGy)	2981.2	3582.9	< 0.005
cord maximum (absolute) (cGy)	4175.0	4587.3	< 0.005
cord maximum (2 cm^3^ volume) (cGy)	3935.9	4422.5	< 0.005
right kidney mean dose (cGy)	474.2	641.8	0.29
left kidney mean dose (cGy)	645.4	966.0	0.26
mean value of (D95^*^/prescription dose) for PTV1 (−)	0.97	0.98	0.70
mean value of (D95^*^/prescription dose) for PTV2 (−)	0.97	0.98	0.48

^*^D95: dose delivered to ≥ 95% of reference volume PTV1 or PTV2.

## Discussion

In this retrospective review of patients with resected high-risk adenocarcinoma of the distal esophagus and GEJ, we describe a novel technique for the delivery of adjuvant chemoRT.

In North America, neoadjuvant chemoRT is the established standard of care for resectable carcinoma of the distal esophagus and GEJ [6-8]. By comparison, the routine use of adjuvant chemoRT is far less common. The published literature regarding adjuvant chemoRT for resected carcinoma of the distal esophagus is characterized mostly by single-institution studies and retrospective reviews, representing a variety of approaches. Our original retrospective study suggested an OS benefit to adjuvant chemoRT over surgery alone, with median overall survival (OS) being extended to 47.5 months with adjuvant chemoRT versus 14.1 months without (p = 0.001) [9]. Irradiation of the anastomosis was associated with a significant improvement in local and regional relapse [10] and reduced recurrence at the anastomotic site [15], implying the importance of local control as a determinant of outcome [16]. Similar benefits of adjuvant chemoRT have been recognized by other authors [17-19].

A comprehensive review of the literature regarding adjuvant chemoRT for resectable carcinoma of the esophagus is provided as an online supplement (Additional file 1).

Adjuvant chemoRT with IMRT-SIB has become our new institutional standard of care for patients with resectable carcinoma of the distal esophagus and GEJ. Our IMRT-SIB technique represents a modern revision of our historical technique [9]. We developed our IMRT-SIB technique for several reasons. In general, IMRT-SIB provides efficiency and convenience relative to 3DCRT. Instead of 2 separate phases of treatment, we need to plan only a single course of treatment, while quality assurance is more efficient than with 2 separate 3DCRT plans. As well, because IMRT had already become established for the treatment of several other disease sites at our institution, we were familiar with its application, delivery and potential clinical and dosimetric benefits, and we were hopeful that IMRT would extend those same benefits to our patients with resected high-risk carcinoma of the esophagus.

Nonetheless, a new technique is not necessarily better simply because it is more technologically advanced. In order to be a true improvement, it must also confer a demonstrable benefit in terms of dosimetry and clinical outcomes, and of course it must be able to do so safely.

**Figure 3:**
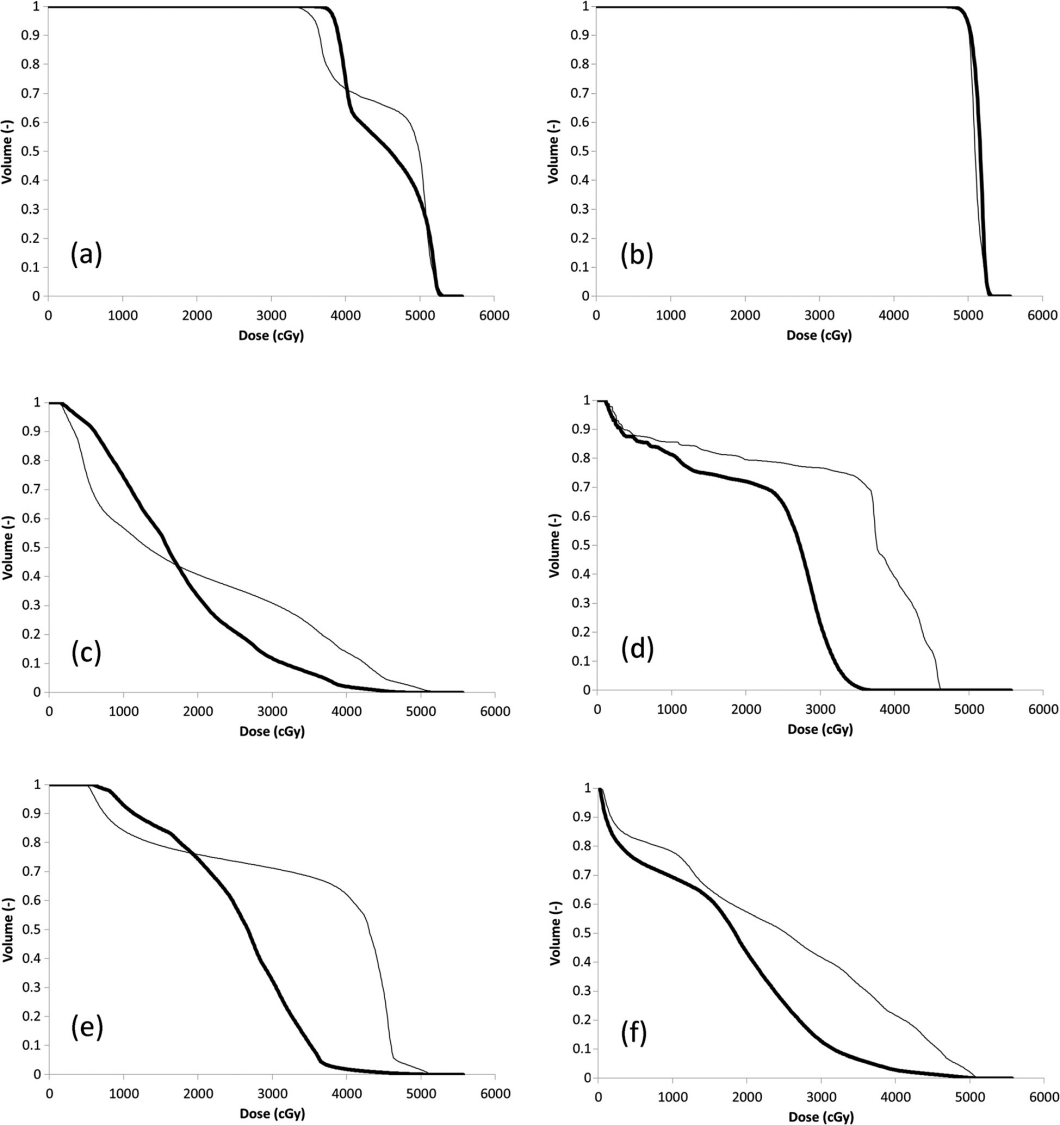
**Example DVH Comparison: IMRT-SIB versus 3DCRTfor: (a) PTV1; (b) PTV2; (c) TL-CTV1; (d) cord; (e) liver; (f) heart (same patient as Figure**1**).** IMRT-SIB (thick lines) and 3DCRT (thin lines) are compared directly for a single illustrative case. IMRT-SIB is more conformal about PTV1 than 3DCRT, with a lower mean dose and a more pronounced dose-gradient from PTV1 to PTV2. There is similar coverage of PTV2. For TL-CTV1 and liver, 3DCRT has better dosimetry in the low-dose region but is worse in the higher-dose region. IMRT-SIB delivers consistently better dosimetry to the heart and the cord over the entire range of dose.

**Figure 4:**
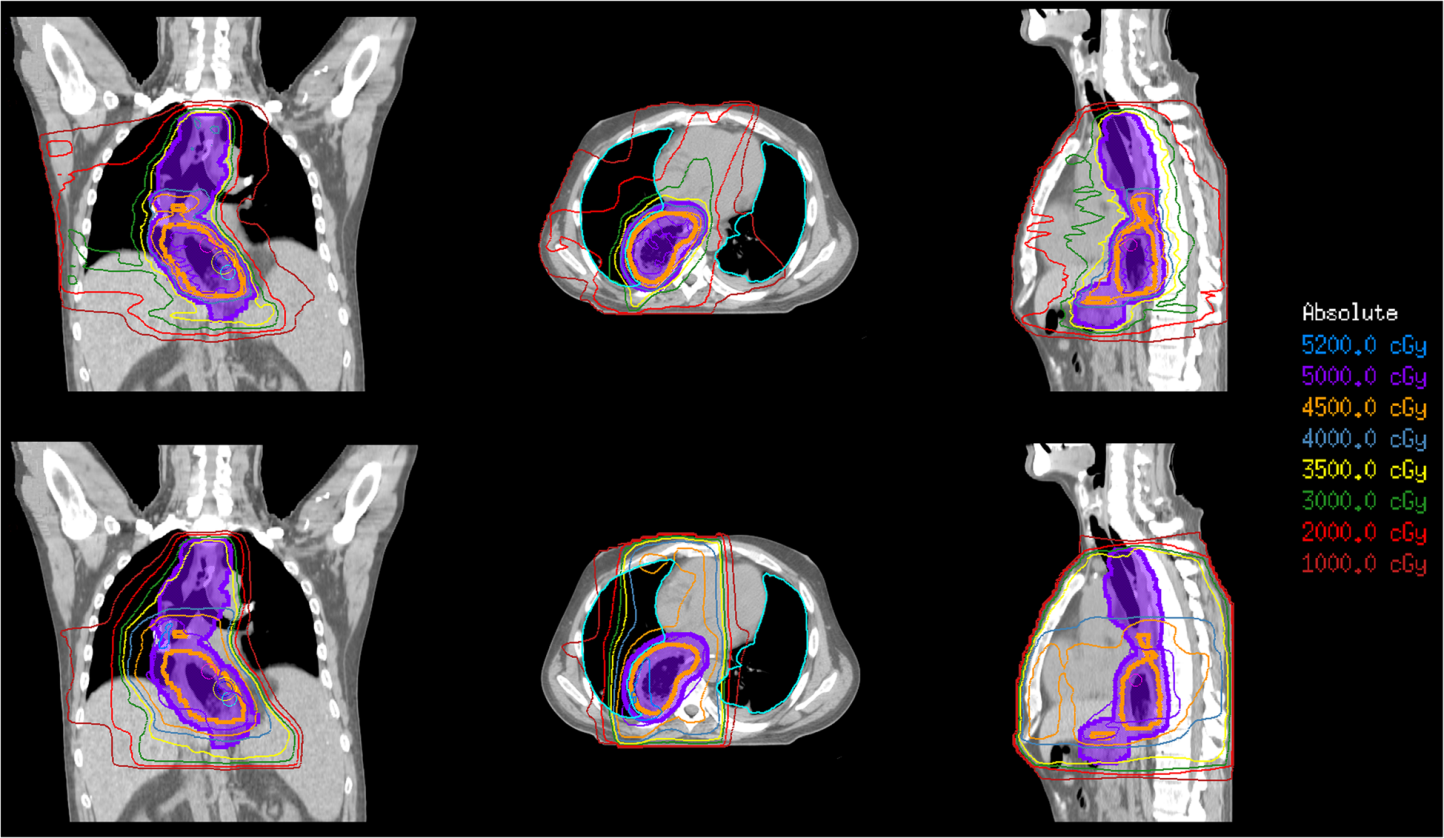
**Example Dosimetric Comparison: IMRT-SIB versus 3DCRT (same patient as Figure**1**).** IMRT-SIB (top) exposes mediastinal structures to generally lower doses than 3DCRT (bottom), conferring considerable potential clinical benefit over 3DCRT.

Our results suggest a considerable dosimetric benefit of IMRT-SIB over 3DCRT. DVH curves (Figure 3) and corresponding dose-distributions (Figure 4) are presented for a single illustrative case. In this example, IMRT-SIB gives improved dosimetry over the entire range of dose for nearly every region of interest except the lung (here represented by TL less CTV1) and the heart. For these 2 structures, 3DCRT produces more favorable dosimetry in the low-dose range than does IMRT-SIB. The benefit of 3DCRT in this range is due to the fact that 3DCRT requires fewer beams, and that such beams typically have distinct field-edges, reducing low-dose scatter compared to IMRT. IMRT-SIB is better in the higher-dose range due to the greater conformality that is possible with IMRT. In general, the larger doses to the mediastinal structures that are delivered using 3DCRT would tend to render 3DCRT less desirable than IMRT-SIB in actual clinical practice.

We compared the observed clinical outcomes of our IMRT-SIB cohort to a previous cohort that we treated using our 3DCRT technique [15]. The relevant results of this comparison are presented in Table 4. Although our outcomes with IMRT-SIB appear to be at least as good as those achieved using 3DCRT, such an interpretation is inherently difficult. We cannot be sure that any observed improvements are due solely to our new technique because comparisons across studies are always difficult, due to such factors as stage migration, improved diagnostic techniques, improved surgical techniques and inter-observer variations in clinical grading and assessment, in addition to the small sample sizes.

**Table 4:** **Comparison of observed clinical outcomes**: **IMRT**-**SIB cohort and published 3DCRT cohort**[15]

		**IMRT-SIB**	**3DCRT**[15]
**Type**	**Outcome**	**(n = 18)**	**(n = 15)**
SURVIVAL	median followup	952 days	570 days (19 months)
	median DFS	not reached	690 days (23 months)
	DFS (1 year)	88%	80%
	DFS (2 years)	82%	44%
	median OS	3 years (36 months)	1.8 years (21 months)
	OS (1 year)	88%	65%
	OS (2 years)	76%	38%
	relapse: local	3 (17%)	0 (0%)
	relapse: distant	7 (39%)	6 (40%)
TOXICITY	pulmonary toxicity (≤90 days)	4 (22%)	2 (15%)
	esophageal toxicity (≤90 days)	9 (50%)	1 (7%)
	pulmonary toxicity (>90 days)	5 (28%)	3 (20%)
	esophageal toxicity (>90 days)	1 (6%)	2 (13%)

One of the potential detriments with our IMRT-SIB technique is the fact that the target volume encompasses the anastomosis, thereby potentially treating a substantial amount of normal lung. It is intriguing that our modern cohort seemed to experience more acute pulmonary toxicity than our historical cohort (Table 4). If the results of our dosimetric comparison (Table 3) are in fact correct, then our 3DCRT technique would have treated the normal lung to a much higher V20 than with IMRT-SIB, correspondingly raising the risk of radiation-induced lung injury. It is possible that the incidence of radiation-induced lung injury was simply under-reported in our original cohort. On the other hand, the significantly increased V10 that we identified for IMRT-SIB over 3DCRT might actually precipitate acute radiation-induced lung injury at a faster rate than would 3DCRT. Interestingly, a recently published series from China did not identify a single case of acute radiation-induced pulmonary toxicity in a cohort of 78 patients receiving adjuvant chemoRT [20]. In that series, 30 of 78 patients were treated to 50 Gy to the lower mediastinum and upper abdomen, while an additional 48 patients were treated to this same dose using an extended field, encompassing the sixth cervical vertebra to the first lumbar vertebra. Although their technique was not thoroughly described, it appears that they delivered all treatment using 3DCRT as opposed to IMRT.

Much of our observed toxicity was either expected (hematologic toxicity in response to chemotherapy) or was clinically insignificant (low-grade pulmonary toxicity). The three most significant observed toxicities were an acute TEF, a late grade 5 aspiration pneumonitis, and a late TEF with simultaneous grade 5 aspiration pneumonitis. The acute TEF occurred within low-dose PTV1, in a patient who required almost-weekly endoscopic dilatation, implying the role of mechanical injury. The late grade 5 aspiration pneumonitis occurred nearly 1 year after the start of RT, in a debilitated patient with widely metastatic recurrent disease. Finally, the late grade 5 aspiration pneumonitis occurred more than 2 years after days after the start of RT, while the TEF was again within low-dose PTV1.

The occurrence of two grade 5 toxicity events is clearly a cause for concern. However, it is not possible to ascertain the significance of this observation from our small, retrospective cohort. We note that grade 5 toxicity was not observed in our previously reported series using 3DCRT [15]. Given the similarity of surgical techniques between our current cohort and our previously reported series, and given the radiobiological similarity between our current and previous dose-fractionation regimes, at the very least we expect IMRT-SIB to be comparable in terms of acute and late toxicity to our previous technique, with comparable survival.

Additional study is therefore required to characterize the efficacy and potential toxicity of our adjuvant IMRT-SIB technique more fully. To this end, we are assessing IMRT-SIB as part of a randomized-controlled clinical trial comparing adjuvant chemoRT and neoadjuvant chemoRT directly, using quality of life as our primary endpoint [21].

## Conclusion

We presented the results of a modern cohort of patients with resected, high-risk adenocarcinoma of the distal esophagus and GEJ, treated with adjuvant chemoRT using IMRT-SIB. Our approach is novel and feasible. Although our approach is associated with acceptable local control and reasonable survival, long-term safety and efficacy still need to be defined. To our knowledge, our study is the first to report the use of IMRT techniques and SIB techniques in this clinical setting. We hope that our experience, as reported here, will be useful to other clinicians when treating this challenging patient population.

## Competing interest

The authors deny any actual or potential conflicts of interest.

## Authors’ contributions

BPY completed the ethics submission, performed the retrospective data collection and analysis and was the primary author. DAP performed the survival analysis calculations and drafted the manuscript. GP and AE performed external beam radiation treatment planning. RAM and RI drafted the manuscript and provided valuable guidance especially regarding the surgical technique. ARD drafted the manuscript. GBR drafted the manuscript and helped to perform the statistical analysis. Edward Yu was senior author and was responsible for drafting the manuscript and for providing valuable clinical and editorial input over the course of this study. All authors read and approved the final manuscript.

## Permissions

Permissions are unnecessary as all content herein was generated by the authors themselves.

## Supplementary Material

Additional file 1 Selected Studies of Adjuvant Chemoradiation for Resected Carcinoma of the Esophagus. Click here for file
