# Supplementary material for: Adjuvant concurrent chemoradiation using intensity-modulated radiotherapy and simultaneous integrated boost for resected high-risk adenocarcinoma of the distal esophagus and gastro-esophageal junction
Source: Radiat Oncol. 2013 Feb 11;8:33. doi: 10.1186/1748-717X-8-33 (PMC3599957; doi:10.1186/1748-717X-8-33)
Supplement: Additional file 1 — Selected Studies of Adjuvant Chemoradiation for Resected Carcinoma of the Esophagus. [file 1748-717X-8-33-S1.docx]

**Table e1 – Selected Studies of Adjuvant Chemoradiation for Resected Carcinoma of the Esophagus**

(OS = overall survival; MS = median survival; DFS = disease-free survival; RFS = recurrence-free survival; TTR = time to recurrence; DMC = distant metastatic control; LRC = locoregional control; FFR = freedom from recurrence; HR = hazard ratio; RR = relative risk; SCC = squamous-cell carcinoma; adeno = adenocarcinoma; C = chemotherapy; RT = radiation therapy; chemoRT = chemoradiation; S = surgery; bid = *bis in die* = twice daily; qd = *quant in die* = daily; 5FU = 5-fluorouracil; IHC = immunohistochemistry)

| **Study** | **Year** | **Clinical** | **Outcomes and Conclusions** |
| --- | --- | --- | --- |
| **Oita Medical University, Japan [**[**1**](#_ENREF_1)**]**  Saito et al. | 1993 | - 35 patients; stage 2-4; histology not specified - adjuvant RT 50 Gy - concurrent cisplatin, vindesine, pepleomycin | - results compared to 26 historical controls - 5-yr OS: 30.9 ± 9.4% (chemoRT); 5.1±4.8% (controls)   **→ adjuvant chemoRT improves OS** |
| **Cleveland Clinic [**[**2**](#_ENREF_2)**]**  Adelstein et al. | **1997** | - phase 2 study - 72 patients (48 adeno, 24 SCC) - accelerated fractionation RT - pre-op RT: 45Gy (1.5 Gy bid) in 3 weeks - surgery 4-6 weeks later - post-op RT: 24Gy (1.5Gy bid) (if residual disease) - chemo: concurrent cisplatin-5FU (during both phases of RT) - conformal RT (IMRT not used) | - no patient completing induction therapy and surgery experienced LRR - 4-yr: RFS 49%, OS 44% - 27% no residual disease at surgery - 18% died perioperatively   **→ results encouraging despite toxicity and perioperative mortality** |
| **St. Luke's, Chicago [**[**3**](#_ENREF_3)**]**  Ebie et al. | **1997** | - phase 1 RT dose-escalation study - 25 patients: 15 adeno; 10 SCC (19 lower third; 6 middle third) - chemo: concurrent cisplatin-5FU 4 cycles - RT: various schemes: 45 Gy (1.5 Gy bid); 40 Gy (2 Gy qd); 50Gy (2Gy mixed bid and qd); all over 3 weeks; conformal RT (IMRT not used) | - median followup 42 months - DFS 58% (1yr), 30% (2yrs); OS 58% (1yr), 32% (2yrs) - median OS 19 mos; median DFS 19 mos; LR ≤ 16%; DM 46% - 2-yr OS: 47% (2 Gy bid); 10% (1.5 Gy bid) (p=ns) - 2 treatment-associated deaths at 2 Gy bid - suggestion of better survival for node-negative patients (p = 0.25)   **→ adjuvant chemoRT improves OS** |
| **Hiroshima University, Japan [**[**4**](#_ENREF_4)**]**  Mukaida et al. | **1998** | - 57 patients; histology “esophageal cancer”; various locations - matched pair analysis: 19 (S+chemoRT); 19 (S+RT); 19 (S) - chemo: concurrent cisplatin-5FU (up to 4 cycles) - RT: approximately 40 Gy (up to 60Gy if positive margin) - RT technique not described | - 5-yr OS 25.2% (S+chemoRT); 18.9% (S+RT); 15.8% (S only) - 5-yr RR 66.7% (S+chemoRT); 54.5% (S+RT); 46.2% (S only); (p = ns)   **→ adjuvant chemoRT does not improve prognosis over surgery alone** |
| **Cleveland Clinic [**[**5**](#_ENREF_5)**]**  Adelstein et al. | **2000** | - esophagus or GEJ; T3, N1 or M1A; 40 pts - 40 patients: 25 adeno; 12 SCC; 3 other - same surgery and accelerated RT regimen as [[2](#_ENREF_2)] - concurrent chemo: (paclitaxel+5FU) versus (cisplatin-paclitaxel) - comparison to 45 historical controls - conformal RT (IMRT not used) | - 3yrs: OS 30%, LRC 81%, DM control is 44% - profound leukopenia & unplanned hospitalization more common with paclitaxel   **→ no advantage to paclitaxel over 5FU, with increased toxicity** |
| **University of Western Ontario [**[**6**](#_ENREF_6)**]**  Bedard et al. | **2001** | - retrospective review - S+chemoRT (38 pts) versus S only (28 pts, historical controls) - ~70% adeno in each group; mostly distal/GEJ in each group - RT: 36Gy/18 to elective volume; then 14Gy/7 to tumor bed - cisplatin-5FU, ±epirubicin - 3D conformal treatment planning | - DFS 10.6 mos (S), 10.2 mos (chemoRT) - LRR 35% (S), 13% (chemoRT) - MS 14.1 mos (S), 47.5 mos (chemoRT) (p = 0.001) - RR mortality (chemoRT) 0.35 (p=0.007)   **→ adjuvant chemoRT prolongs OS over surgery alone** |
| **INT 0116 [**[**7**](#_ENREF_7)**]**  Macdonald et al. | **2001** | - randomized controlled study - resected gastric adenoCA (20% GEJ) - 281 patients (S+chemoRT) vs. 275 patients (S alone) - RT: 45Gy/25 - chemo: concurrent 5FU + leucovorin - 3D conformal treatment planning | - HR (death) 1.35 (1.09-1.66; p=0.005) - HR (relapse) 1.52 (1.23-1.86; p<0.001) - results not stratified for disease site (GEJ not reported separately)   **→ adjuvant chemoRT associated with better OS** |
| **Cleveland Clinic [**[**8**](#_ENREF_8)**]**  Rice et al. | **2003** | - retrospective review - matched-pair analysis   risk-unadjusted analysis:   - S+chemo+RT (n=31); S (n=52) - 26 adeno; 5 squamous   propensity-matched analysis:   - S+chemoRT (n=20); S (n=20) - 43 adeno; 9 squamous - RT (all cases): 50.4-59.4 Gy; 1.8 Gy/fraction - chemo: concurrent cisplatin-5FU | risk-unadjusted:   - S + chemoRT: MS 28 mos; OS 68±8.4% (1yr), 44±9% (4yrs) - S alone: MS 14 mos; OS 60±6.8% (1yr), 17±5.6% (4yrs) (p = 0.05) - median TTR 25 mos (S+chemoRT) versus 13 mos (S alone) (p = 0.15) - median RFS 22 mos (S+chemoRT) versus 11 mos (S alone) (p = 0.04)   propensity-matched:   - S + chemoRT: MS 28 mos; OS 60±11% (1yr), 44±11.3% (4yrs) - S alone: MS 15 mos; OS 65±10.7% (1yr), 0% (4yrs) (p = 0.05) - median TTR 25 mos (S+chemoRT) versus 13 mos (S alone) (p = 0.04) - median RFS 22 mos (S+chemoRT) versus 10 mos (S alone) (p = 0.02)   **→ adjuvant chemoRT doubles OS, time to recurrence, RFS over surgery** |
| **Shimane Medical University [**[**9**](#_ENREF_9)**]**  Tachibana et al. | **2003** | - 45 patients, all SCC, randomized after S - chemo (n=23); chemoRT (n=22) - chemo: concurrent cisplatin-5FU - RT: 50Gy/25 to tumor bed - RT technique not described - IHC performed for tumoral p53 | - chemo: OS 100% (1yr), 63% (3yr), 38% (5yr) - chemoRT: OS 80% (1yr), 58% (3yr), 50% (5yr) (p=0.97) - p53 negative (n=18): OS 75% (3yrs) and 64% (5yrs) - p53 positive (n=27): OS 44% (3yrs) and 26% (5yrs) (p=0.03)   **→ chemoRT does not improve OS over chemo alone**  **→ chemoRT may improve OS over chemo if p53 expression negative** |

| **Canberra Hospital [**[**10**](#_ENREF_10)**]**  Hughes et al. | **2004** | - 45 patients treated as part of INT 0116 or similar local approaches - 29 gastric, 12 GEJ, 4 distal esophagus - 78% ≥N1; 15.5% had positive margins - RT: 45Gy/25 (same as INT 0116) - conformal RT (IMRT not used) | toxicity:   - overall: G3 (37.8%), G4 (4.4%) - gastrointestinal only: G3 (20%) - hematologic: G3/4 (17.8%) - 7/45 (15.6%) required delay in RT; 2 patients (4.4%) did not receive RT - chemo: dose reduction (31.8%); dose delay (50.0%)   at median followup 16 mos:   - 28 relapses (62.2%); 6 LR alone (13.3%) - 22 deaths (48.9%); 21/22 deaths due to cancer (46.7%)   **→ outcomes were not stratified according to disease location**  **→ INT 0116 protocol is safe and feasible** |
| --- | --- | --- | --- |
| **University of Western Ontario [**[**11**](#_ENREF_11)**]**  Yu et al. | **2004** | - retrospective study - median follow up 30.5 mos - 69 pts: 65% adeno, 35% SCC - T3, T4, ≥N1, ±margin involved - 43 pts: local RT; 26 pts: extended-field RT - RT: 45-50 Gy (elective); 60 Gy (boost) - chemo: (epirubicin+cisplatin-5FU) or (cisplatin-5FU) - conformal RT (IMRT not used) | local RT:   - MS 32.5 mos, OS 60.5% (2yr), OS 29.2% (5yr) - DSS: median 36.4 mos, 66.1% (2yr), 31.9% (5yr)   extended RT:   - MS 27.6 mos, OS 42.5% (2yr), OS 34.6% (5yr) (p = ns) - DSS: median 35.2 mos, 59.7% (2yr), 49.7% (5yr) - anastomotic recurrence: 29% (local RT), 0% (extended) (p = 0.041) - LR as % of all relapses: 74.2% (local RT), 15.4% (extended RT) (p< 0.001)   **→ extended adjuvant chemoRT decreases locoregional relapse** |
| **Mackay Memorial Hospital, Taiwan [**[**12**](#_ENREF_12)**]**  Liu et al. | **2005** | - non-randomized prospective study - 60 patients; T3/4 and N0/1 - adjuvant: chemoRT (n=30) versus RT (n=30) - RT: 55-60 Gy | - OS: 30.9 (27.5-36.4) mos (chemoRT), 20.7 (15.2-26.1) mos (RT) - OS (3 yr): 70.0% (chemoRT); 33.7% (RT alone) (P = 0.003)   **→ adjuvant chemoRT is well tolerated and effective** |
| **Hiroshima University, Japan [**[**13**](#_ENREF_13)**]**  Shimizu et al. | **2005** | - 57 pts; advanced SCC; T2-4, N1, M0 - 14 pts received adjuvant chemoRT - RT: 50Gy/25; RT technique not described - chemo: 5FU+cisplatin (both induction and concurrent) | - matched pair analysis: chemoRT vs S - OS (5 yrs): 35.7% (chemoRT), 28.5% (S) (p = ns) - recurrence (5 yrs): 50% (chemoRT), 57% (S) (p = ns)   **→ combined chemoRT did not improve prognosis** |
| **Cleveland Clinic [**[**14**](#_ENREF_14)**]**  Adelstein et al. | **2007** | - mature results of previous accelerated RT study [[5](#_ENREF_5)] - 93 pts enrolled - before surgery: 30Gy at 1.5 Gy/fraction bid - after surgery: 30Gy at 1.5 Gy/fraction bid - concurrent cisplatin-5FU | - median follow up 50 mos (34-72 mos) - 3yrs: OS 27.9%, FFR 30.5%, DM control 32.4%, LRC 86% - improved FFR with: lower stage, SCC, pathologic response   **→ accelerated multimodality program is feasible**  **→ overall survival disappointing due to distant metastases** |

| **Cleveland Clinic [**[**15**](#_ENREF_15)**]**  Adelstein et al. | **2009** | - mature results of [[8](#_ENREF_8)] - 50 high-risk patients - location: mid 4 (86%); lower 10 (20%); GEJ 36 (72%) - node positive 43 (86%); adeno 7 (14%) - RT: 50.4-59.4 Gy ; 1.8 Gy/fraction | - 4yrs: OS 51%, FFR 50%, DMC 56%, LRC 86% - outcomes with chemoRT are better than historical results of S alone - earlier pathologic stage predicts for better outcome   **→ postoperative concurrent chemoRT is feasible** |
| --- | --- | --- | --- |
| **University of Western Ontario [**[**16**](#_ENREF_16)**]**  Yu et al. | **2009** | - 15 patients; adeno (n=5), SCC (n=10); resected T3/4, N0/1 - prospective accrual; pilot study; - RT: 30.6Gy/17 (elective volume), then 19.8Gy/11 (tumor bed) - 3D conformal technique - 2 cycles epirubicin-cisplatin-5FU (before RT) - 2 cycles cisplatin-5FU (during RT) | - median follow up 19 months (3.5-53.4 months) - 20% experienced chemotherapy delay during RT - recurrence in 40% (6/15); median time to relapse 24 mos - no recurrences at anastomosis - median DFS 23 months; median OS 21 mos   **→ adjuvant extended-volume chemoRT is feasible and safe** |
| **Wenzhou Medical College [**[**17**](#_ENREF_17)**]**  Wu et al. | **2009** | - planning study to investigate the role of adjuvant chemoRT - 52 pts with esophagectomy - stage 2/3 esophageal carcinoma - RT: 50Gy in 25 fractions - concurrent cisplatin-paclitaxel (2 overlapping cycles) | - grade 3 or 4 toxicity in 54% - 29/52 pts alive at median follow-up 23.5 months; MS 37.2 months - 53.8 % (28/52) recurrence at median follow up - distant metastasis was dominant mode of failure - at 2 yrs: LRC 60%; DM-free survival 71%; RFS 47% - OS 82% (1-yr), 42% (3yrs)   **→ novel postoperative chemoRT is tolerable and has promising outcomes** |
| **Cleveland Clinic [**[**18**](#_ENREF_18)**]**  Rodriguez et al. | **2010** | - phase II study added gefitinib (G) to previous regimen [[14](#_ENREF_14)] - 80 pts; T3, N1, or M1A esophagus or GEJ; ≥ 80% adeno - retrospective comparison with 93 historical controls - same accelerated RT regimen as [[14](#_ENREF_14)] - before surgery: 30Gy at 1.5 Gy/fraction bid - after surgery: 30Gy at 1.5 Gy/fraction bid - concurrent cisplatin + 5FU + gefitinib with RT - maintenance gefitinib 2 years - mixed 2D and 3D treatment planning - comparison to historical controls [[14](#_ENREF_14)] | - 3yr: OS 42% (chemoRT+G), 28% (chemoRT) (p=0.06) - 3yr: DMC 40% (chemoRT+G), 32% (chemoRT) (p=0.33) - 3yr: LRC 76% (chemoRT+G), 77% (chemoRT) (p=0.74) - toxicity: rash (53%), diarrhea (55%)   **→ G did not worsen toxicity during chemoRT; maintenance G difficult**  **→ (chemoRT+G) gives superior OS not solely related to decreased DMC** |

| **University of Miami [**[**19**](#_ENREF_19)**]**  Solomon et al. | **2010** | - 2 merged patient databases (Florida) - 3,378 pts with resected adeno (636 esophageal; 2742 gastric) - individual benefits of adjuvant RT and adjuvant chemo were assessed   of 636 patients with esophageal cancer:   - 76% lower esophagus; 31.4% nodal spread - RT 38.8 %; chemo 42.6%; combined: not stated - stratification: overall, localized, regional & distant disease - RT technique not described | esophageal adeno  MS (overall): adjuvant chemo/RT no benefit  MS (localized): adjuvant chemo/RT no benefit  MS (regional): 18.6 mos (RT), 13.5 mos (no RT) (p=0.007)  MS (regional): 20.0 mos (chemo), 13.0 mos (no chemo) (p<0.001)  MS (distant): 14.0 mos (RT), 7 mos (no RT) (p=0.246)  MS (distant): 21.0 mos (chemo), 5.0 mos (no chemo) (p<0.001)  HR death (regional): 0.656 (RT) (p=0.01); 0.535 (chemo) (p<0.001)  gastric adeno  MS (overall): 23.8 mos (RT); 17.0 mos (no RT) (p<0.001)  MS (overall): 20.9 mos (chemo); 17.5 mos (no chemo) (p=0.012)  MS (localized): no benefit MS for chemo or RT  MS (regional): 22.6 mos (RT), 12.3 mos (no RT) (p<0.001)  MS (regional): 21.6 mos (chemo), 11.2 mos (no chemo) (p<0.001)  MS (distant): 15.5 mos (RT), 6.7 mos (no RT) (p<0.001)  MS (distant): 10.6 mos (chemo), 5.1 mos (no chemo) (p<0.001)  HR death (regional): 0.603 (RT) (p<0.001); 0.629 (chemo) (p<0.001)  **→ adjuvant RT and adjuvant chemo each improve survival for regional presentations of adenocarcinoma of the esophagus and stomach but not for localized presentations** |
| --- | --- | --- | --- |

| **Nanjing Medical University [**[**20**](#_ENREF_20)**]**  Lv et al. | **2010** | - phase 3 prospective randomized-controlled trial - 238 patients, all SCC (~30% distal esophagus), stage 2 or 3 - patients randomized to 3 groups: - preop chemoRT (n=80) - postop chemoRT (n=80) - surgery alone (S) (n=80) - primary endpoint PFS; secondary endpoint OS - preop RT: 40 Gy (2 Gy fractions); no boost reported - postop RT: 40 Gy then boost 10 Gy (2 Gy fractions) - concurrent cisplatin-paclitaxel - median follow up 45 mos | all patients   - PFS (preop): 89.3% (1yr); 61.3% (3yr); 37.5% (5yr); 18.1% (10yr) - PFS (postop): 89.1% (1yr); 61.1% (3yr); 37.2% (5yr); 17.8% (10yr) - PFS (S): 84.5% (1yr); 49.3% (3yr); 25.9% (5yr); 6.2% (10yr)   (p=0.0151)   - OS (preop): 91.3% (1yr); 63.5% (3yr); 43.5% (5yr); 24.5% (10yr) - OS (postop): 91% (1yr); 62.8% (3yr); 42.3% (5yr); 24.4% (10yr) - OS (S): 87.5% (1yr); 51.3% (3yr); , 33.8% (5yr); 12.5% (10yr)   (p=0.0176)   - OS & PFS: no differences between preop and postop (p>0.05) - LRR: 11.3% (preop), 14.1% (postop), 35% (S) (p<0.05) - no significant differences in observed toxicity (p>0.05)   subset: patients proceeding to radical resection   - median PFS 48 mos (preop); 61 mos (postop); 39.5 mos (S) (p=0.0331) - OS 56.5 mos (preop); 72 mos (postop); 41.5 mos (S) (p=0.0153) - no differences between preop and postop chemoRT (p>0.05)   **→ preoperative or postoperative chemoRT improve OS and PFS over S alone** |
| --- | --- | --- | --- |
| **Ankara [**[**21**](#_ENREF_21)**]**  Uncu et al. | **2010** | - resected gastric (n=57) and GEJ (n=9) adenocarcinoma - stage 3 (n=38), stage 4 (n=27) - 45 Gy in 25 fractions - boost 10Gy in 5 fractions (if margin positive) - conformal RT only (IMRT not used) - pre-RT cisplatin-5FU-folate & post-RT cisplatin-5FU-folate | - median follow-up 15 mos (6-36 mos) - median DFS: 18 mos (13.9-22.0 mos) - median OS: 19 mos (15.2-22.8 mos)   **→ regimen of adjuvant chemoRT is effective and tolerable** |
| **Los Angeles County Database [**[**22**](#_ENREF_22)**]**  McKenzie et al. | **2011** | - review of Los Angeles County Cancer Surveillance Program - 2233 patients identified - stage 1-3 esophageal cancer - 38% adeno; 55% SCC; 48% lower esophagus - chemoRT (n=645); chemoRT+S (n=286) - comparison: chemoRT versus chemoRT+S - RT technique not described | Entire cohort   - MS 13.1 mos - MS 25.2 mos (chemoRT+S) vs 12.3 mos (chemoRT) (p<0.001) - HR death 0.66 (0.56-0.77)(P<0.001)   adenoCA:   - MS 25.9 mos (chemoRT+S) vs 10.6 months (chemoRT) (p<0.001)   SCC:   - MS 24.5 mos (chemoRT+S) vs 12.8 mos (chemoRT) (p<0.001)   **→ S+chemoRT a significant prognostic factor for improved survival** |

| **Innsbruck Medical, Austria [**[**23**](#_ENREF_23)**]**  Spizzo et al. | **2011** | - phase 2 feasibility study - gastric and GEJ - preop cisplatin-docetaxel, resection, then postop chemoRT - adjuvant RT: 39.6 Gy at 1.8 Gy per fraction; concurrent 5-FU infusion - RT technique not described | - 15/15 (100%) went to surgery - 11/15 (73%) had R0 resection - 6/11 with R0 (54%) received adjuvant chemoRT - 5/6 with R0 and chemoRT disease-free at median 72 months - patients with R0: OS 73% (6yrs)   **→ preoperative chemo, surgery then adjuvant chemoRT is feasible** |
| --- | --- | --- | --- |

REFERENCES

1. Saito, T., et al., *Cisplatin, vindesine, pepleomycin and concurrent radiation therapy following esophagectomy with lymph adenectomy for patients with an esophageal carcinoma.* Oncology, 1993. **50**(4): p. 293-7.

2. Adelstein, D.J., et al., *Use of concurrent chemotherapy, accelerated fractionation radiation, and surgery for patients with esophageal carcinoma.* Cancer, 1997. **80**(6): p. 1011-20.

3. Ebie, N., et al., *Integration of surgery in multimodality therapy for esophageal cancer.* American Journal of Clinical Oncology, 1997. **20**(1): p. 11-5.

4. Mukaida, H., et al., *Clinical evaluation of adjuvant chemoradiotherapy with CDDP, 5-FU, and VP-16 for advanced esophageal cancer.* The Japanese journal of thoracic and cardiovascular surgery : official publication of the Japanese Association for Thoracic Surgery = Nihon Kyobu Geka Gakkai zasshi, 1998. **46**(1): p. 11-7.

5. Adelstein, D.J., et al., *Does paclitaxel improve the chemoradiotherapy of locoregionally advanced esophageal cancer? A nonrandomized comparison with fluorouracil-based therapy.* Journal of Clinical Oncology, 2000. **18**(10): p. 2032-9.

6. Bedard, E.L., et al., *The role of surgery and postoperative chemoradiation therapy in patients with lymph node positive esophageal carcinoma.* Cancer, 2001. **91**(12): p. 2423-30.

7. Macdonald, J.S., et al., *Chemoradiotherapy after surgery compared with surgery alone for adenocarcinoma of the stomach or gastroesophageal junction.* New England Journal of Medicine, 2001. **345**(10): p. 725-30.

8. Rice, T.W., et al., *Benefit of postoperative adjuvant chemoradiotherapy in locoregionally advanced esophageal carcinoma.* Journal of Thoracic & Cardiovascular Surgery, 2003. **126**(5): p. 1590-6.

9. Tachibana, M., et al., *Postoperative chemotherapy vs chemoradiotherapy for thoracic esophageal cancer: a prospective randomized clinical trial.* Eur J Surg Oncol, 2003. **29**(7): p. 580-7.

10. Hughes, B.G., et al., *Audit of postoperative chemoradiotherapy as adjuvant therapy for resected gastroesophageal adenocarcinoma: an Australian multicentre experience.* ANZ journal of surgery, 2004. **74**(11): p. 951-6.

11. Yu, E., et al., *Is extended volume external beam radiation therapy covering the anastomotic site beneficial in post-esophagectomy high risk patients?* Radiotherapy and oncology : journal of the European Society for Therapeutic Radiology and Oncology, 2004. **73**(2): p. 141-8.

12. Liu, H.-C., et al., *Esophagectomy for locally advanced esophageal cancer, followed by chemoradiotherapy and adjuvant chemotherapy.* World Journal of Gastroenterology, 2005. **11**(34): p. 5367-72.

13. Shimizu, K., et al., *Clinical evaluation of low-dose cisplatin and 5-fluorouracil as adjuvant chemoradiotherapy for advanced squamous cell carcinoma of the esophagus.* Hiroshima Journal of Medical Sciences, 2005. **54**(3): p. 67-71.

14. Adelstein, D.J., et al., *A phase II trial of accelerated multimodality therapy for locoregionally advanced cancer of the esophagus and gastroesophageal junction: the impact of clinical heterogeneity.* American Journal of Clinical Oncology, 2007. **30**(2): p. 172-80.

15. Adelstein, D.J., et al., *Mature results from a phase II trial of postoperative concurrent chemoradiotherapy for poor prognosis cancer of the esophagus and gastroesophageal junction.* Journal of Thoracic Oncology, 2009. **4**(10): p. 1264-9.

16. Yu, E., et al., *Postoperative extended-volume external-beam radiation therapy in high-risk esophageal cancer patients: a prospective experience.* Curr Oncol, 2009. **16**(4): p. 48-54.

17. Wu, S.D., X; Zhang, P; Xie, C; Zhang, X; Jin, Z, *Phase II study of postoperative chemoradiotherapy for esophageal carcinoma.* Journal of Clinical Oncology, 2009. **27**(suppl): p. abstr e15606.

18. Rodriguez, C.P., et al., *A phase II study of perioperative concurrent chemotherapy, gefitinib, and hyperfractionated radiation followed by maintenance gefitinib in locoregionally advanced esophagus and gastroesophageal junction cancer.* Journal of Thoracic Oncology, 2010. **5**(2): p. 229-35.

19. Solomon, N.L., et al., *Does chemoradiotherapy improve outcomes for surgically resected adenocarcinoma of the stomach or esophagus?* Annals of Surgical Oncology, 2010. **17**(1): p. 98-108.

20. Jin Lv, X.-F.C., Bin Zhu, Lv Ji, Lei Tao, Dong-Dong Wang, *Long-term efficacy of perioperative chemoradiotherapy on esophageal squamous cell carcinoma.* World J Gastroenterol, 2010. **16**(13): p. 1649-54.

21. Uncu, D., et al., *Adjuvant bi-weekly combination of cisplatin, infusional 5-fluorouracil and folinic acid followed by concomitant chemoradiotherapy with infusional fluorouracil for high risk operated gastric and gastroesophageal junction adenocarcinoma.* Asian Pacific Journal of Cancer Prevention: Apjcp, 2010. **11**(6): p. 1493-7.

22. McKenzie, S., et al., *Improved outcomes in the management of esophageal cancer with the addition of surgical resection to chemoradiation therapy.* Annals of Surgical Oncology, 2011. **18**(2): p. 551-8.

23. Spizzo, G., et al., *Preoperative chemotherapy with cisplatin and docetaxel followed by surgery and clip-oriented postoperative chemoradiation in patients with localized gastric or gastroesophageal junction adenocarcinoma: results from a phase II feasibility study.* Annals of Surgical Oncology, 2011. **18**(3): p. 677-83.
